# Pure Squamous Cell Carcinoma of the Duodenum

**DOI:** 10.1155/2015/714640

**Published:** 2015-02-16

**Authors:** Muharrem Battal, Ozgur Bostancı, Tulay Basak, Kinyas Kartal, Feza Ekiz

**Affiliations:** ^1^General Surgery Department, Sisli Etfal Research Hospital, Istanbul, Turkey; ^2^Pathology Department, Sisli Etfal Research Hospital, Istanbul, Turkey; ^3^General Surgery Department, Istanbul Medical Faculty, Istanbul University, Istanbul, Turkey

## Abstract

Primary carcinomas of the small intestine are extremely rare neoplasms. Most of these are adenocarcinomas. Primary squamous cell carcinoma (SCC) of small intestine is exceptionally rare with only occasional case reports in the literature. We report here a surgically treated patient with squamous cell carcinoma arising from duodenal diverticula in the third part of the duodenum.

## 1. Introduction

Primary carcinomas of the small intestine are extremely rare neoplasms accounting for only 0.1–1.3% of all gastrointestinal tract neoplasms [1]. Most of these are adenocarcinomas and located in the second part of the duodenum. Primary squamous cell carcinoma (SCC) of small intestine is exceptionally rare with only occasional case reports in the literature [2, 3]. We report here a surgically treated patient with squamous cell carcinoma arising from duodenal diverticula in the third part of the duodenum.

## 2. Case Report

**Figure 1:**
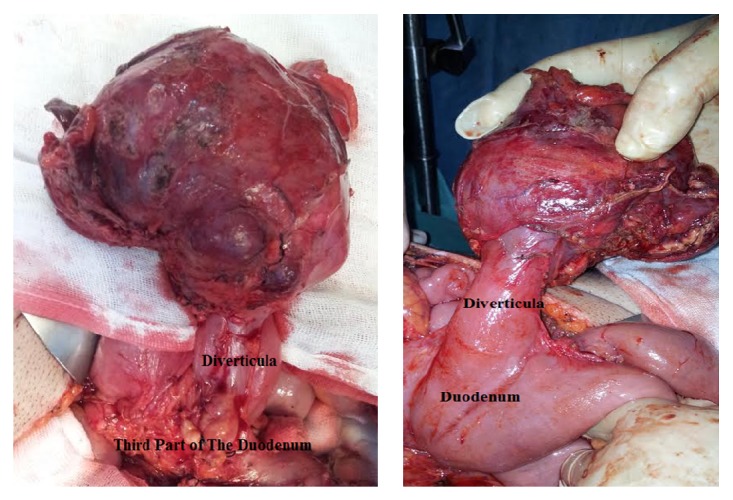
The macroscopic view of the mass.

A 39-year-old young man was admitted to our hospital with epigastric pain, lack of appetite, weakness, and vomiting. He had a weight loss of 8 kg in one-month period. All of the laboratory assessments including tumor markers were at normal levels. During endoscopic examination mild antral gastritis is observed. There were no endoscopically pathological findings evaluated in the first and the second parts of the duodenum. A CT scan of the abdomen showed a mass in the third part of the duodenum (Figure 1). The diameter of mass was 60 × 62 × 55 mm. There was no evidence of metastasis in the thorax CT. After accomplishing the preoperative readiness the patient was operated on. During operative observation, we detected a diverticula located on the third part of the duodenum. And at the tip of the diverticula's there was 8 cm mass. The diverticula were resected from the far end with stapler device. The patient was discharged at the postoperative fourth day with no complications.

**Figure 2:**
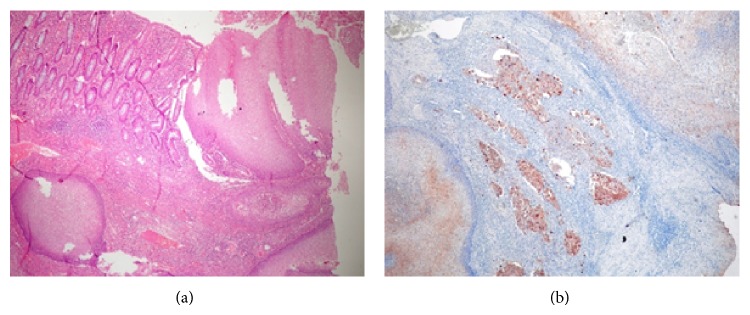
(a) Duodenal mucosa and squamous cells are seen together, stained with H&E, 40-fold magnification. (b) Invasive foci stained with cytokeratin staining, 40-fold magnification.

The pathological examination revealed a mass with 9 × 7 × 8 cm diameters. Histological examination showed a well-differentiated squamous cell carcinoma arising from the small intestinal epithelium. Common squamous metaplasia focuses were seen in the glandular epithelium of the diverticulum. Regional lymph nodes and resection margins were free of tumor. Immunohistochemically, the tumor was CK positive and negative for S-100, Vimentin, and TTF-1 (Figure 2). The Ki-67 index was 31%.

## 3. Discussion

The squamous cell carcinoma of the duodenum is exceedingly rare and only occasional case reports are seen in the literature [4, 5]. The pathogenesis of squamous cell carcinoma of the duodenum is still unknown. They may arise in congenital anomalies such as duplications and diverticula [6]. Most of the squamous cell carcinomas of the duodenum are metastatic tumors from other solid organs such as cervix or lung [7]. The optimal treatment and the prognosis of squamous cell carcinoma of the duodenum are elusive because of the rarity of this condition [8].

The clinical presentation of squamous cell carcinoma of the duodenum is similar to that of other duodenal tumors. Imaging studies such as abdominal CT scan, magnetic resonance imaging and/or magnetic resonance cholangiopancreatography, and ERCP can reveal the tumor but histological diagnosis may not always be possible. The optimal treatment and the prognosis of squamous cell carcinoma of the duodenum are elusive because of the rarity of this condition. Surgery is the corner stone in the management of the disease [9]. Due to the small number of cases available in the literature, the effects of chemoradiotherapy on the survival and recurrence are unknown.

Histopathological examination is the most reliable way to distinguish the primary and metastatic tumors of the gastrointestinal tract tumors. Pathogenetic differences between metastatic and primary tumors are also helpful for the clinicians in the differential diagnosis. Many different hypotheses have been demonstrated for elucidation of the pathogenesis of SCC cases detected in the stomach and duodenum such as nests of ectopic squamous cells, the proliferation of uncommitted mucosal basal cells into squamous cells, squamous metaplasia secondary to chronic mucosal damage, squamous differentiation in a preexisting adenocarcinoma, and multipotent stem cells in the gastrointestinal mucosa [10]. Our case supports the literature by the development of SCC from the ectopic mucosa of the duodenal diverticulum.

We found precious little case reports about duodenal SCC. Most of these cases were in the second part of the duodenum near the ampulla of Vater and these patients were predominantly treated nonsurgically. We represent an exceptional case with duodenal squamous cell carcinoma in the third part of the duodenum that is treated surgically. According to our knowledge this is the third case in the transverse duodenum described in the medical literature [2, 9]. The other two cases were inoperable at the time of the diagnosis. So this is the first case of the squamous cell carcinoma of the third duodenum which is treated surgically.

After the operation, oncologists stated that there was no need for adjuvant chemotherapy treatment. Ten months after the surgery, the patient had no evidence of recurrence or metastasis. Patient continues the daily life in a healthy way. Further documentation of this rare tumor will provide a better understanding of its pathogenesis and management for optimal survival rates.
